# Chemical Recycling of Bio-Based Epoxy Matrices Based on Precursors Derived from Waste Flour: Recycled Polymers Characterization

**DOI:** 10.3390/polym17030335

**Published:** 2025-01-26

**Authors:** Lorena Saitta, Sandro Dattilo, Giuliana Rizzo, Claudio Tosto, Ignazio Blanco, Francesca Ferrari, Gloria Anna Carallo, Fabrizio Cafaro, Antonio Greco, Gianluca Cicala

**Affiliations:** 1 Department of Civil Engineering and Architecture, University of Catania, Via Santa Sofia 64, 95125 Catania, Italy; giuliana.rizzo@unict.phd.it (G.R.); claudio.tosto@unict.it (C.T.); iblanco@unict.it (I.B.); gianluca.cicala@unict.it (G.C.); 2 CNR-IPCB, Via Paolo Gaifami 18, 95126 Catania, Italy; 3 Department of Engineering for Innovation, University of Salento, Via per Monteroni, 73100 Lecce, Italy; francesca.ferrari@unisalento.it (F.F.); gloriaanna.carallo@unisalento.it (G.A.C.); fabrizio.cafaro@unisalento.it (F.C.); antonio.greco@unisalento.it (A.G.)

**Keywords:** recyclable thermosets, circular economy, chemical recycling, bio-based epoxy resins, organic waste, bio-content, green epoxidation

## Abstract

This study aims to investigate the chemical recycling of two different fully recyclable bio-based epoxy matrices based on epoxidized precursors derived from waste flour. The key for their recyclability relies on the use of a cleavable hardener. In fact, the latter contains a ketal group in its chemical structure, which is cleavable in mild acetic conditions, so allowing for the breakage of the cured network. The recyclability was successfully assessed for both the two investigated formulations, with a recycling process yield ranging from 80 up to 85%. The recycled polymers presented a *T*_g_ up to 69.0 ± 0.4 °C, determined by mean of DMA and DSC analysis. Next, the TGA revealed that the thermal decomposition of the specimens primarily occurred around 320 °C and attributed to the breaking of C–O and C–N bonds in cross-linked networks. In the end, the chemical characterizations were carried out by mean of Py-GC/MS, MALDI-TOF-MS and FT-IR ATR. In fact, these analyses allowed for investigating how the recycled polymer’s structure changed, starting from the initial epoxy systems. These insights on their chemical structure could further allow for identifying re-use strategies in accordance with a circular economy approach.

## 1. Introduction

Among the thermoset class of polymer materials, the epoxy resins are the most used one. This is justified by their cheap cost even by maintaining excellent properties, like simple processability, outstanding chemical resistance and durability, minimal thermal expansion, in addition to good dielectric, thermal and mechanical properties [1,2,3,4,5]. For this reason, epoxy resins are widely employed in many industrial engineering fields, like automotive, aerospace, wind energy, sport, offshore and naval, to realize fiber-reinforced composite materials for high-performance applications. Moreover, they also found application in the electronic and optical field, where the transparency is required [6,7,8] and the precise refractive index value should be determined [9]. In fact, this class of materials present very interesting properties, i.e., high strength, durability, low thermal expansion, extremely high strength-to-weight ratio, high modulus, corrosion resistance, large degree of freedom of design and ease of fabrication [3,4,5]. This trend is confirmed by a forecasting analysis run by the Global Epoxy Composite Market Size Report (2021–2028), which assess that the global epoxy composite market (valued at USD 28.40 billion in 2021) is projected to grow at a compound annual growth rate (CAGR) of 8.3% from 2021 to 2028 [10]. Despite the use of this class of material being widespread in the aforementioned sectors, which need lightweight materials to improve efficiency, environmental considerations must be made in relation to their use [11,12]. Indeed, since epoxy resins are three-dimensional (3D) cross-linked structures [13,14,15] encompassing an epoxide main chain reacting together with a curing agent to create permanent covalent intermolecular bonds [16], their recyclability is generally complicated [17]. So, the two main used disposal options for fiber-reinforced epoxy composite materials, once they have reached their End-of-Life (EoL), are incineration and landfills [18,19,20]. However, several sociotechnical pressures are driving towards the development of more sustainable disposal solutions, such as recycling routes, because landfills are becoming banned in EU countries [21,22] due to the negative environmental impacts. Even the incineration strategy is not environmentally friendly [23]. Otherwise, a lack of recycling facilities may cause a barrier to the increased use of epoxy-based composite materials [24]. Therefore, research activities that aim to develop suitable strategies for epoxy resins recycling are of imminent urgency.

In agreement with the state-of-the-art, the main recycling strategies proposed for the fiber-reinforced epoxy resin composites are the mechanical, thermal and chemical recycling technologies [21,25,26]. Even though the thermal recycling procedure, such as pyrolysis, is classified as a high technology readiness level (TRL 8) by reaching capacities ranging between 1000 and 2000 tons [21,27], the obtained recycled products, which is the fibers, present degraded properties. Meanwhile, the matrices are lost due to degradation caused by the very high temperatures reached during the process (from 350 °C up to 800 °C) [28,29]. Similarly, the mechanical recycling approach, which involves crushing, grinding, milling and shredding techniques, only allows for recovering fibers that have reduced properties with complete matrix degradation [21,30]. Conversely, the chemical technologies exploiting mild chemical degradation as a recycling strategy allow for the recovering of undamaged high-value natural and synthetic fibers, together with the matrix. In fact, the latter can be properly fully depolymerized to obtain either reusable monomers or oligomers [16,31,32,33,34,35,36,37]. So, even though a TRL of 4 was achieved for this route [27], both academia and industry should work together to enhance this class of disposal strategies. In particular, they should develop thermosets characterized by the presence of labile cleavable bonds, thus allowing thermoset depolymerization under mild conditions via bond cleavage [32,33,36,38,39,40]. Moreover, reusable recycled products should be simultaneously obtained to follow a cradle-to-cradle (C2C) approach in line with a circular economy (CE) philosophy. This means aiming in the epoxy-based composite materials field to remodel an exhausted product for generating a new life cycle, hence transforming it into something that can be infinitely reused rather just being recycled with a deterioration of properties as final result [41].

Aside from what has been discussed so far, an additional environmental concern regarding the usage of fiber-reinforced epoxy resin composites is related to the use of petroleum-based raw materials for the epoxy monomer synthesis. This is due to the use as prepolymer in epoxy resin of diglycidyl ether of bisphenol A (DGEBA), obtained through the reaction in the presence of a basic catalyst between epichlorohydrin and bisphenol A (BPA), with the latter being both reprotoxic and toxic for living organisms [42,43]. Furthermore, in the near future we must deal with the uncertainty related to finite petrochemical resources [4,44]. For all these reasons, researchers must aim to produce bio-based epoxy resins, showing thermo-mechanical properties comparable with already commercially available DGEBA ones by replacing petroleum-based monomers with raw materials derived from renewable resources [45,46,47,48,49,50,51,52].

In this work, we have extended the sustainable chemical recycling procedure for epoxy resins relying on the Recyclamine^TM^ technology (patented from Connora^®^ Technology, US Patent 2013/0245204 A1). However, an optimized approach already developed by the authors in [32] was used in this study for recycling two different bio-based epoxy formulations. The latter relies on the combined use of a commercial bio-based epoxy prepolymer having 28% of biocarbon content derived from pine oils with an epoxidized monomers derived from a treatment with UV/ozone radiation of waste flour, which was recovered from the processing waste of pasta factories [53,54]. Thus, the key for the novelty of this work lies in synergistically integrating two distinct approaches: developing bio-based epoxy blends with the purpose of increasing as much as possible the bio-content of the epoxy resin and designing them to enable chemical recycling as a disposable route.

The present work aims to thermally and chemically characterize developed thermoset systems and the relative recycled polymers to find, in the next future, valuable reuse strategies. This allows for achieving a truly C2C approach, similarly to what has been already performed in previous works with the commercial thermoset. In the latter-mentioned studies, the recycled polymer was used as toughening agent of epoxy resin by adding it in different content to the virgin resin [36]; as a sustainable material suitable for extrusion-based 3D printing processes [55] and electrospinning [56]; and for synthesizing new matrices [32]. In the end, it should be highlighted that the disposable route proposed in this study, i.e., chemical recyclability, was proved to also be efficient in terms of cost, as also confirmed by a recent study which examined the advantages of Recyclamine™ use from a life cycle cost (LCC) perspective [57].

## 2. Materials and Methods

### 2.1. Materials

In this study, the recyclability of two different epoxy resin formulations was assessed. The first was obtained by properly mixing a bicomponent system, made of an industrial scale bio-based epoxy prepolymer and a fully recyclable amine hardener, with an epoxidized waste flour (EWF) while the second was prepared by properly mixing the EWF with the fully recyclable amine hardener.

In detail, the used bio-based epoxy prepolymer, named PolarBear (part A), is characterized by 28% of biocarbon content, determined according with the ASTM D6866-22 B Method (AMS) TOC coming from pine oils. Furthermore, the key factor for the amine recyclability, named Recyclamine^TM^ R*101, is the presence of a cleavable ketal group under mild acid condition, thus allowing for the epoxy resin’s network breakage. The bio-based epoxy prepolymer was purchased by R*CONCEPT (Barcelona, Spain), while the Recyclamine^TM^ R*101 was provided by the Aditya Birla Group (Mumbai, India). In the end, the EWF was synthetized by following a patented procedure [58] reported elsewhere [53]. Basically, the latter relied on a simultaneous UV and ozone treatment for 5 h of waste flour derived from waste of pasta factories.

The chemical recycling procedure of the two epoxy systems was performed by using pure acetic acid purchased from VWR International S.r.l., Milan, Italy, and ammonium hydroxide (28.0–30.0% NH_3_ basis) purchased from Sigma-Aldrich (Merk Life Science S.r.l., Milan, Italy).

### 2.2. Epoxy Resin Systems Formulation

The recyclability via a chemical recycling process was assessed for the two different developed epoxy systems. The details for each of them are reported in Table 1. More deeply, the first formulation, labeled as EWF50_A15, was selected as the most performing in a previous study carried out by the authors [54]. It was obtained by mixing 50%wt of PolarBear with 50%wt of EWF and then using a content of the Recyclamine^TM^ R*101 equal to 15 phr (per hundred resin). Conversely, the second formulation, named EWF100_A22, was obtained by exploiting as part A of the formulation only the EWF and then by adding a content of the Recyclamine^TM^ R*101 equal to 22 phr. At first, the two considered formulations were mechanically mixed and then degassed by applying a vacuum at room temperature. After this, the two formulations were poured in silicon molds, cured at room temperature for 24 h, and next post-cured at 150 °C for 2 h.

### 2.3. Chemical Recycling Process

The recyclability of the two prepared epoxy resin formulations was assessed by exploiting a chemical recycling procedure already optimized by the authors [32]. In detail, the epoxy resin samples were fragmented in small pieces, 10 g were dissolved in 300 mL of acetic acid aqueous solution, made of 75%vol of acetic acid and 25%vol of distilled water, at 80 °C for 90 min. The resulting opalescent solution was roto-evaporated up to a volume of 75 mL, at a temperature of 60 °C and a pressure of 60 mbar. In this way, it was possible to recover most of the aqueous acetic acid solution and to obtain a more concentrated solution. The latter was neutralized by using 150 mL of ammonium hydroxide solution, made of distilled water and ammonium hydroxide (28–30% NH_3_ basis) by using a 1:1 volume ratio. A whitish material started to precipitate, which was the recycled polymer. It was next recovered by centrifugation at 3000 rpm, for 5 min. The supernatant was removed and the recycled polymer filtered and dried at 50 °C for 24 h.

**Figure 1:**
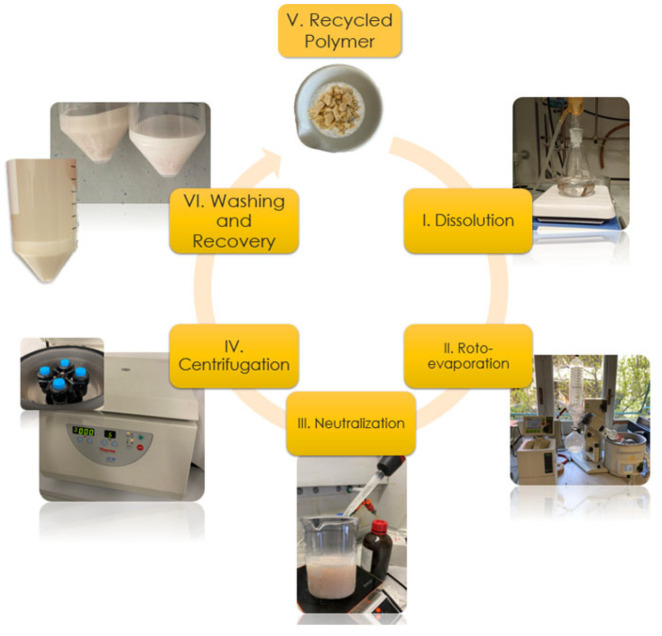
Chemical recycling process schematization.

The above-described procedure was exploited both for the EWF50_A15 and EFW100_A22 systems. So, the recovered recycled polymers were labeled as rEWF50_A15 and rEFW100_A22, respectively. The chemical recycling procedure is schematized in Figure 1.

In the end, for both the two analyzed epoxy systems, the recycling process yield was evaluated using the following equation:

(1) $$yield(\%)=\frac{W_{f}^{\left(rE\right)}}{W_{i}^{\left(E\right)}\;}\times 100$$

with $W_{f}^{\left(rE\right)}$ being the final weight of the recycled polymer (once dried) and $W_{i}^{\left(E\right)}$ the initial weight of the cured epoxy system.

### 2.4. Recycled Polymers: Characterization Techniques

#### 2.4.1. Dynamic Mechanical Analysis (DMA)

The glass transition temperature (*T*_g_) of the developed thermosets (EWF50_P50_A15 and EWF_A20) and the correspondent recycled polymers (rEWF50_P50_A15 and rEWF_A20) were determined by running a dynamic mechanical analysis (DMA). The latter test was performed by means of a dynamic mechanical thermal analyzer TRITEC2000 (Triton Technology, Leicestershire, UK). As the two recycled polymers were in powder form, they were tested by using the pocket DMA approach. It is a technique used for testing powders in the pharmaceutical industry [59]. In detail, 0.35 g of polymer powder were put into a standard stainless-steel pocket, purchased from Triton Technology (Leicestershire, UK), and pressed to obtain a uniform thickness. Once the sample’s temperature was stabilized at 25 °C, it was heated up to 100 °C with a heating rate of 2 °C/min, at 1 Hz of frequency and in single cantilever deformation mode. The latter is a standard method for the polymers characterization through the glass transition [60]. For each tested sample, the tanδ versus temperature curve was plotted, and the *T*_g_ was determined in correspondence of the peak. Three samples for each recycled polymer were tested, so the considered response was expressed as mean ± st. deviation.

#### 2.4.2. Differential Scanning Calorimetry (DSC)

The calorimetric measurements of the two developed thermoset systems (EWF50_A15 and EFW100_A22) and the relative recycled polymers (rEWF50_P50_A15 and rEWF_A20, respectively) obtained at the end of the chemical recycling process were carried out by mean of a Mettler DSC1 instrument from Mettler Toledo (Greifensee, Switzerland). For each run test, about 4–7 mg of sample were put within sealed aluminum crucibles with a volume equal to 40 μL. Once the analysis started, each sample was heated from 25 °C up to 250 °C at a heating rate of 20 °C/min in air.

#### 2.4.3. Termogravimetric Analysis (TGA)

The thermal stability of both the two developed thermoset systems (EWF50_A15 and EFW100_A22) and the corresponding recycled polymers (rEWF50_P50_A15 and rEWF_A20, respectively) was investigated by thermogravimetric analysis (TGA). The latter were run using a Mettler TGA1 instrument from Mettler Toledo (Greifensee, Switzerland). Each test was carried out on 7–15 mg of sample, in air, with a heating rate of 10 °C/min and a temperature interval ranging from 50 °C up to 800 °C. For each sample, both the mass loss versus temperature and the derivative thermogravimetric versus temperature curves were determined.

#### 2.4.4. Pyrolysis–Gas Chromatography–Mass Spectrometry (Py-GC/MS)

Pyrolysis (TD, PY) coupled with GC/MS was performed on the two developed thermoset systems (EWF50_A15 and EFW100_A22) and the relative recycled polymer, i.e., rEWF50_P50_A15 and rEWF_A20, respectively. For each analysis, a small amount of sample (about 0.1 mg) was placed in a crucible and pyrolyzed by a Multi-Shot Pyrolyzer (EGA/PY-3030D, Frontier Labs, Koriyama, Fukushima, Japan). Pyrolysis products were separated and identified by a GC system GC-2020 (Shimadzu Corporation, Kyoto, Japan), coupled with a triple quadrupole mass spectrometry detector and electronic ionization (70 eV) Mass Detector TQ8040 (Shimadzu Corporation). The gas chromatography was equipped with Ultra Alloy^®^ Metal Capillary Column (Frontier Labs Ltd., Koriyama, Fukushima, Japan) stationary phase 5% di-phenyl-methylpolysiloxane, with an inner diameter of 250 μm, a film thickness of 0.25 μm and a length of 30 m. Interfaces of Py-GC and GC/MS were kept at 300 °C and 250 °C, respectively. One step of PY-GC-MS was performed for each sample at a PY temperature of 600 °C. The temperature program of the GC oven was the following: 50 °C for 1 min, increased up to 100 °C at 30 °C/min; 100 °C for 5 min, from 100 °C to 300 °C at 10 °C/min and finally it was fixed at 300 °C for 10 min. Helium was selected as carrier gas, with a flow rate of 1.78 mL/min. The split ratio was 1/50 of the total flux. A control run was carried out before each pyrolysis analysis by placing the empty crucible in the furnace and performing pyrolysis at the same conditions mentioned above.

#### 2.4.5. Matrix-Assisted Laser Desorption/Ionization Time-of-Flight Mass Spectroscopy (MALDI-TOF-MS)

MALDI TOF analysis was carried out by using a 4800 MALDI TOF/TOF™ Analyzer (Applied Biosystem, Framingham, MA, USA), equipped with a Nd:YAG laser (wavelength of 355 nm) characterized by <500 ps pulse, a repetition rate of 200 Hz and working in positive-ion mode.

MALDI mass spectra were recorded in reflector mode. For masses in the range *m*/*z* 200–5000 Da, the mass resolution and accuracy of the MALDI spectra were about 8.000 (full width at half maximum, FWHM) and 1–10 ppm, respectively. Samples preparation was performed by dissolving the recycled polymer (rEWF50 _A15 and rEWF100_A20), with a concentration of 10 mg/mL, and the matrix 2–5 dihydroxy benzoic acid (DHB) (0.1 mmol) in acetic acid water solution (75% *v*/*v*). Appropriate volumes of samples and matrix solutions were mixed to obtain 1:1, 1:2 and 2:1 ratios (sample/matrix *v/v*). Then, 1 μL of each sample was deposited onto the MALDI sample holder and dried at room temperature to allow matrix crystallization.

#### 2.4.6. FT-IR Analysis

The Fourier Transform Infrared Spectroscopy (FT-IR) spectra of both recycled polymers, i.e., rEWF50_A15 and rEWF100_A20, were collected by using a Perkin Elmer Spectrum 100 UATR (Waltham, MA, USA) spectrometer in attenuated total reflectance (ATR) mode. The absorption bands were recorded exploiting a resolution of 4 cm^−1^ and 16 scans, into a range varying between 4000 and 650 cm^−1^. Next, the OMNIC 6.3.1. software was used for data analysis. FT-IR analysis allowed for investigating how the recycled polymer’s structure changed, starting from the initial epoxy systems. The FT-IR analysis related to the latter formulation were carried out elsewhere by the authors [53].

## 3. Results and Discussion

### 3.1. Chemical Recycling Process Results and Process Yield

**Figure 2:**
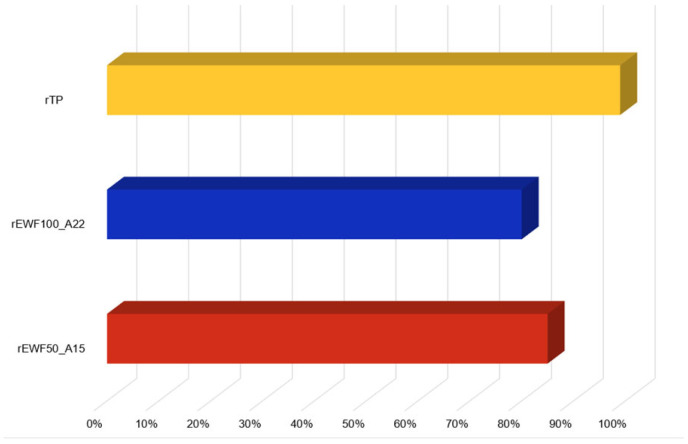
Comparison among the recycled products obtained from the chemical recycling process: rTP (yellow) [32], rEWF100_A22 (blue) and rEWF50_A15 (red).

The recycled polymers obtained from the two epoxy formulations were identified as rEWF50_A15 (derived from EWF50_A15) and rEWF100_A20 (derived from EWF100_A22). Both of them were whitish compound in the solid state, thus simply crushable into powder. Furthermore, the recycled polymer rEWF50_P50_A15 was recovered with a process yield of 85%, while the recovery process yield for rEWF_A20 was slightly lower, i.e., equal to 80%. Thus, despite the original epoxy matrix, already treated by the authors [32], was modified by adding variable quantities of epoxidized waste flour, the used chemical recycling process allowed for the full depolymerization of the two cured networks as well. However, the epoxidized waste flour addition within the formulation caused a decrease in the process yield, since it was equal to 99% for the formulation not containing the epoxidized waste flour [32]. The comparison among the recycling process yield for the obtained recycled products is reported in Figure 2.

### 3.2. Chemical and Thermo-Mechanical Properties of the Recycled Product

**Figure 3:**
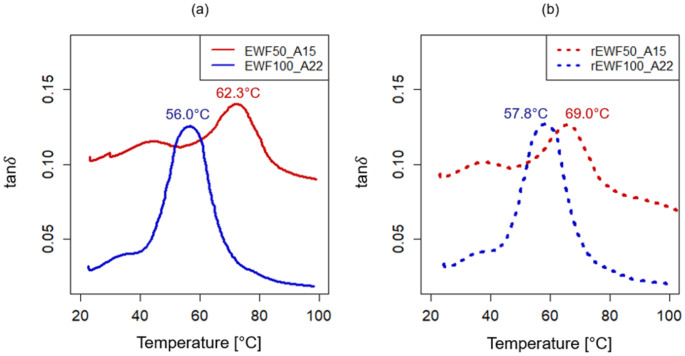
DMA tanδ versus temperature plots: (**a**) EWF50_A15 (red curve) and EWF100_A22 (blue curve). (**b**) Recycled polymers: rEWF50_A15 (dotted red curve) and rEWF100_A22 (dotted blue curve).

The developed epoxy systems and the recycled polymers obtained from the recycling process were characterized both by DMA and DSC analysis to determine their *T*_g_. The tanδ versus temperature curves collected for each analyzed sample are reported in Figure 3. According to the obtained results, the recycled polymer rEWF50_A15 showed a *T*_g_ value of 69.0 ± 0.4 °C, while for the rEWF100_A22 it was equal to 57.8 ± 0.2 °C (see Figure 3b). Thus, a reduction of 10% and 32% was found for the rEWF50_A15 and rEWF100_A22, respectively, when compared to the recycled thermoplastic obtained for the epoxy system not containing the EWF (equal to 76 °C) [32]. However, the two recycled polymers showed a higher temperature than a recycled product obtained starting from a bio-based epoxy resin, cured using an aromatic disulfide cross-linker with diacid, and varying the epoxy precursor among different epoxidized soybean oils cured by 2,2′-dithiodibenzoic acid [61].

**Figure 4:**
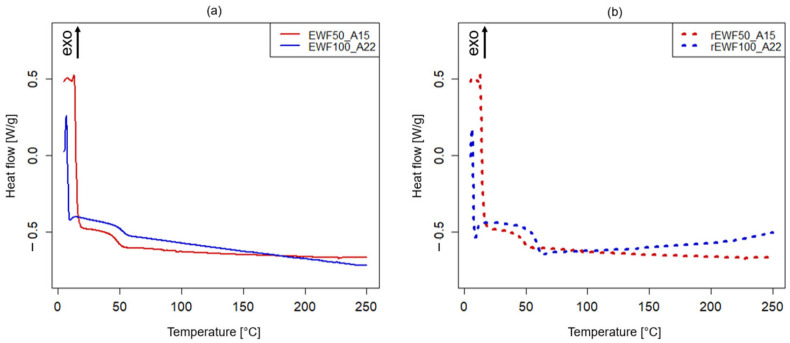
DSC thermograms of (**a**) epoxy resins: EWF50_A15 (red curve) and EWF100_A22 (blue curve); (**b**) recycled polymers: rEWF50_A15 (dotted red curve) and rEWF100_A22 (dotted blue curve).

Moving on, the DSC thermograms for both the initially developed thermoset systems and the corresponding recycled materials are reported in Figure 4. Here, a *T*_g_ value of 63.1 °C was found for the rEWF50_A15 sample, while it was 8% lower for the rEWF100_A22 recycled polymer, i.e., equal to 58.1 °C (see Figure 4b). The obtained results are consistent with the DMA results, even though the *T*_g_ values determined through the latter analysis are higher due to the faster time scales and the mechanical nature of the measurement. In fact, the latter relies on thermal events changes, such as heat flow *C*_p_, when dealing with DSC analysis; conversely, the DMA measurements are based on the viscoelastic properties and mechanical modulus transitions, which emphasize relaxation processes requiring more thermal energy [62]. Furthermore, it must be highlighted that the recycled polymers obtained at the end of the chemical recycling process (rEWF50_A15 and rEWF100_A22) have an added value when compared to the initial thermoset systems in terms of the determined *T*_g_ value, since an increase of about 35% and 12% was found when compared to the initial formulated epoxy systems. In fact, in accordance with the DSC analysis, the *T*_g_ resulted to be equal to 46.6 °C for EWF50_A15 and 51.8 °C for the EWF100_A22 (see Figure 4a). A similar trend was also confirmed from the DMA results, being the *T*_g_ value lower for the initial epoxy systems (see Figure 3a) when compared to the correspondent recycled polymers (see Figure 3b).

**Figure 5:**
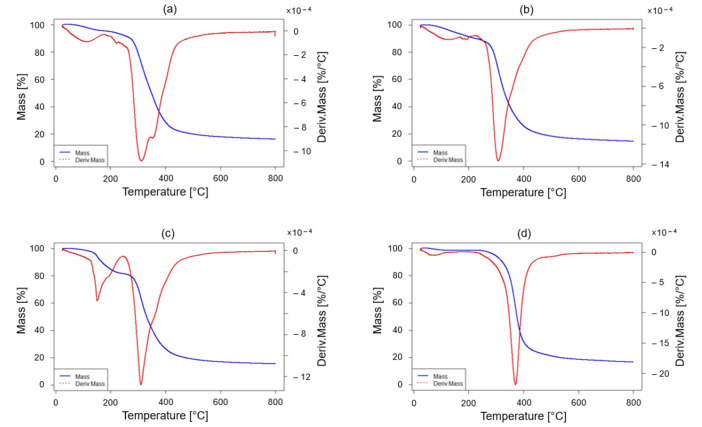
TGA thermograms of the epoxy resin and recycled polymers: (**a**) EWF50_A15; (**b**) EWF100_A22 (blue curve); (**c**) rEWF50_A15; (**d**) rEWF100_A22.

In Figure 5 are reported the thermograms related to the TGA of the two developed thermoset systems, that is, EWF50_A15 and EFW100_A22, and the relative recycled polymers, i.e., rEWF50_A15 and rEWF_A20, respectively. Here, it can be inferred that neither the variation in the initial thermoset systems nor the performed chemical process affected the thermal stability of the polymer materials. In fact, in each investigated scenario the thermal decomposition of the specimens occurred in the main region at about 320 °C. The latter phenomenon can be associated with the thermal scission of C–O and C–N bonds in cross-linked networks derived from the reaction between epoxy groups and hardeners [63]. However, it must be noted that the thermogram related to rEWF50_A15 (see Figure 5c) presented an initial mass loss of about 30% at 150 °C as well. Thus, the recycled thermoplastic derived from the thermoset system also containing the PolarBear epoxy resin (EWF50_A15) had a lower onset degradation temperature with respect to the recycled polymer derived from the thermoset containing only flour (EFW100_A22). This latter finding can be explained as follows. When amines are cleaved in acetic acid solutions, these may leave behind trapped acetic acid, byproducts from the neutralization cleavage reaction, and amine derivatives that may not have been fully removed after processing. These small volatile molecules typically begin to evaporate or thermally degrade at relatively low temperatures, such as around 180 °C, resulting in a significant mass loss. Indeed, for example, ammonium acetate decomposes between 100 and 200 °C, releasing water, acetic acid, or ammonia gases. This means that rEWF50_A15 recycled product trapped a higher quantity of impurities during the performed chemical recycling process selected as disposable solution. For this reason, a drying process performed at temperatures higher than 50 °C may represents a good strategy for obtaining a purer recycled product. In the end, it must be taken into account that the addition of epoxidized monomers derived from a treatment with UV/ozone radiation of waste flours within the already developed epoxy system made of only PolarBear and Recyclamine^TM^ R*101 implied that the obtained recycled product is characterized by a lower thermal stability [32]. Hence, thermal degradation temperatures of 320 versus 400 °C were found for the recycled polymers derived from thermosets with and without the addition of epoxidized monomers derived from waste flours, respectively.

**Figure 6:**
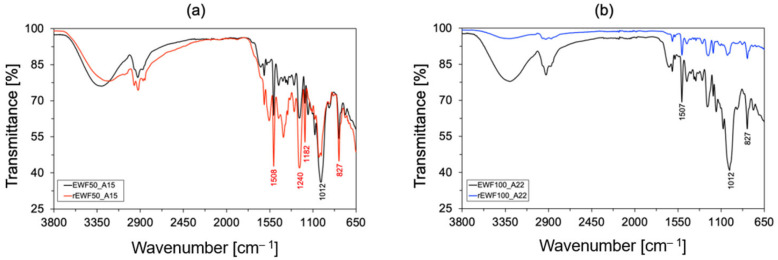
FT-IR acquired spectra for the recycled polymer: (**a**) EWF50_A15 (black curve) vs. rEWF50_A15 (red curve); (**b**) EWF100_A22 (black curve) vs. rEWF100_A22 (blue curve).

To have more insight on the chemical nature of generated products in the acid attack of the resins, the recycled samples were characterized by FT-IR, MALDI-TOF and PY-GC-MS. A comparison between the chemical structures of EWF50_A15 and EWF100_A22, before and after the recycling process, were evaluated by FT-IR spectroscopy. The results are represented in Figure 6a,b, respectively. In the fingerprint regions of Figure 6a, strong peaks at around 1500 cm^−1^ can be observed in all spectra associated with the asymmetrical stretching of nitro groups (N–O) of cleavable amines used as curing agents. Similarly, intense band at 1240 cm^−1^ and a peak at 1180 cm^−1^ can be associated with the alkyl aryl ether groups characterizing both the resins. Peaks with lower intensity were reported in the fingerprint region of EWF100_A22 which can be associated with moieties of functionalized phenols deriving from waste flour. Broad band with medium intensity at 1407 cm^−1^ was reported for the recycled rEWF50_A15 thermoplastic linked to the stretching of O–H group in alcohols. This comes from the breakage of acetals in the curing agents during the recycling process. The mechanism was reported in a previous paper of the authors [32]. The peak at 1012 cm^−1^ were reported as well in both spectrums before the recycling process, associated with the stretching of the alkyl aryl ether groups. These are generally correlated to the presence of PolarBear, characterized by bisphenol structure [32]. A similar peak at 1034 cm^−1^ was reported in the spectrum of rEWF100_A22, associated as well with the bending of C–H bonded to the ether group. The absence of a peak between 920 and 900 cm^−1^ in both spectra confirms as well the full curing of the pristine thermosets due to the absence of epoxy ring stretching. Typical peaks at 827 cm^−1^ associated with the stretching of the aromatic system were evaluated, once again, before and after the recycling process of both resins. Broad bands between 3300 and 3200 cm^−1^ were typically associated with hydroxylic stretching, while small peaks at 2920 cm^−1^ were associated with the C–H stretching of the aromatic ring.

**Figure 7:**
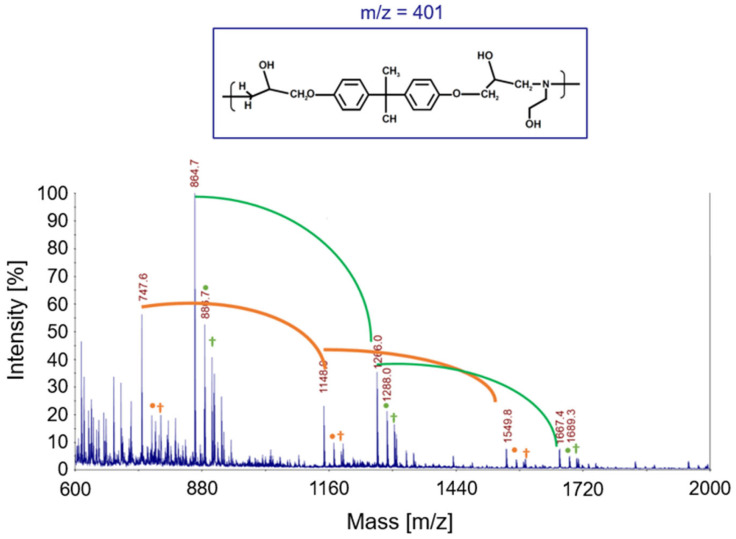
MALDI-MS spectra of the recycled products rEWF50_A15.

**Figure 8:**
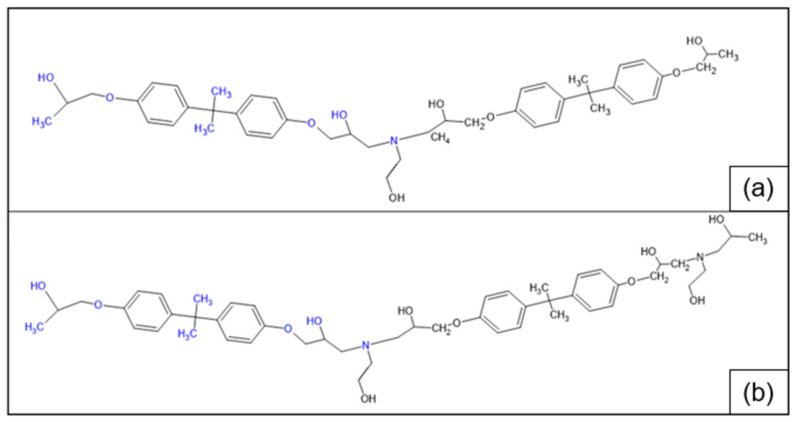
Structure with m/z 746 (**a**) and 864 (**b**) as adducts with proton ⁄ ⁄, sodium •• and potassium †† in the MALDI spectra of the recycled products rEWF50_A15 (see Figure 7).

**Figure 9:**
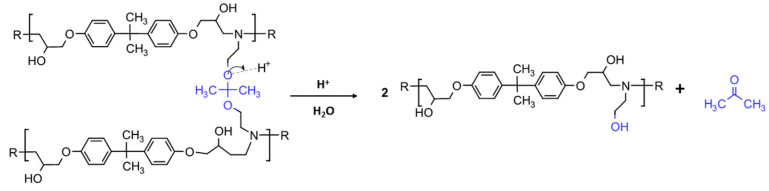
Acid degradation mechanism of epoxy resin.

In Figure 7, the MALDI-TOF mass spectrum of the recycled polymers is represented, while the structure of the most intense peaks are assigned in Table 2. Two families of peaks can be identified in the spectrum ions at *m*/*z* 747.6 + n401 and *m*/*z* 864.7 + n401 corresponding to protonated ions of compounds, whose structures are shown in Figure 8. These ions are accompanied by their sodium and potassium adducts which are labeled with circles (••) and crosses (††), respectively. Since the MALDI ionization occurs without any fragmentation, the products identified in the spectrum have been certainly generated by the acid-induced degradation of the amine cross-linking agent of DGEBA/polar-based epoxy resin, according to the mechanism shown in Figure 9. The mechanism involves the acid-catalyzed degradation of a ketal. One of the ether oxygen atoms in the ketal is protonated by H^+^, increasing its susceptibility as a leaving group. Water, present in the system, attacks the resulting carbocation to form a hemiketal intermediate. Ultimately, the reaction yields two alcohol molecules and a ketone.

The MALDI-TOF spectrum of the sample with flour contains no useful information for its characterization, most likely due to the high molecular weight of the flour matrix, and it is omitted here for brevity.

**Figure 10:**
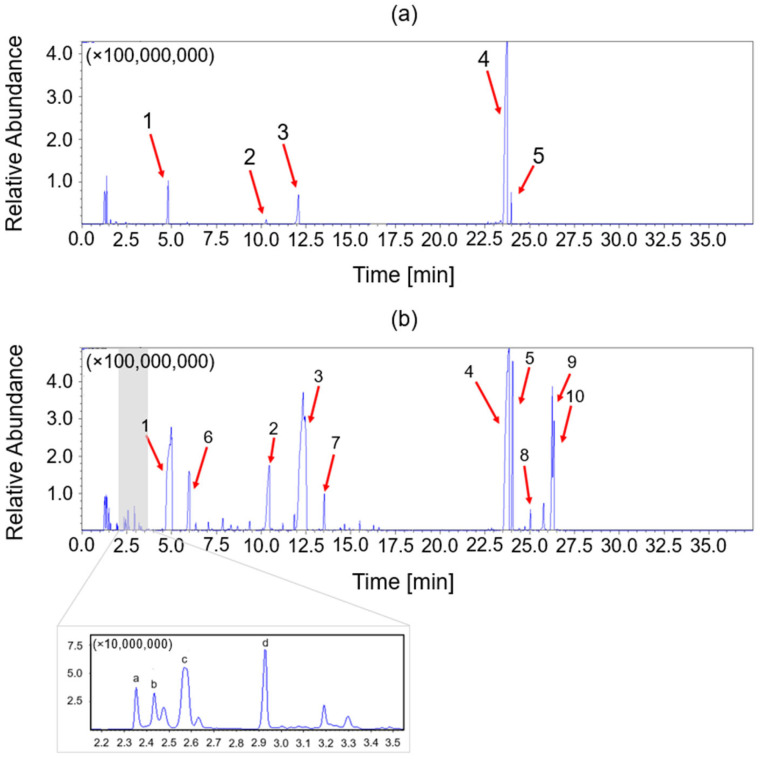
Pyrolysis-GC/MS acquired chromatogram of recycled epoxy system EWF50_A15 (**a**) and recycled product rEWF50_A15 (**b**) decomposition in helium.

Figure 10 shows the pyrolysis chromatograms of epoxy system EWF50_A15 (Figure 10a) and its recycled sample rEWF50_A15 (Figure 10b). In both cases, the most intense peak is due to bisphenol A (Table 2), characteristic of DGEBA-based systems. The pyrolysis spectrum in Figure 10a shows less peaks with respect to the recycled epoxy resins (Figure 10b), most likely due to the lower degradation temperature of the latter sample that generates more amounts of pyrolysis products. Peaks 1–10 represent pyrolysis compounds with phenol and bisphenol A units generated by the pyrolysis of products of the acid hydrolysis of epoxy/polar resins. In Figure 10b, there is another series of peaks appearing at a low retention time (labeled a–d) which have been identified as degradation products containing nitrogen atoms such as pyrrole, pyrazole, oxazolidine and oxazine (see Table 2). These products have probably been formed by the thermal degradation of amine cross-linker. For the systems EWF100_A22 and rEWF100_A22 spectra were collected and characterized by similar peaks, which are reported in Figures S1 and S2 of the Supplementary Materials. However, the latter present other uninterpretable peaks, likely due to the high degree of cross-linking.

## 4. Conclusions

This study focuses on investigating chemical recyclability as a sustainable disposable strategy for fully recyclable bio-based epoxy matrices based on epoxidized precursors derived from waste flour. In detail, two different formulations were investigated:

- EWF50_A15, containing 50%wt of PolarBear with 50%wt of EWF, and Recyclamine^TM^ R*101 equal to 15 phr;
- EWF100_A22, obtained by directly mixing only the EWF with a content of Recyclamine^TM^ R*101 equal to 22 phr.

The recycled polymers obtained by employing a chemical recycling strategy on the aforementioned thermoset formulations were identified as rEWF50_A15 and rEWF100_A22, respectively. These recycled products were achieved with a recycling process yield equal to 85% for the EWF50_A15 blend, while it was slightly lower for the EWF100_A22 formulation, i.e., equal to 80%. Thus, the here presented experimental study permitted to integrate the use of sustainable raw materials with the recyclability of products at the end of their lifecycle. In fact, the former consists of the epoxy prepolymer PolarBear containing 28% of the bio-content derived from pine oils and the use of epoxidized precursors derived from waste flour.

Furthermore, the obtained recycled products from the chemical recycling procedure were fully characterized both in terms of thermal and chemical analysis. In detail, rEWF50_A15 presented a *T*_g_ of 69.0 ± 0.4 °C, while rEWF100_A22 was of 57.8 ± 0.2 °C. This thermal property was determined by means of both DMA and DSC analysis. Next, the TGA revealed that the thermal decomposition of the specimens primarily occurred around 320 °C, attributed to the breaking of C–O and C–N bonds in the cross-linked networks. However, it was discovered that the rEWF50_A15 recycled polymer presented a lower onset degradation temperature with respect to the rEWF100_A22. In fact, it also showed an initial mass loss of about 30% at 150 °C, which is due to the fact that the rEWF50_A15 recycled product trapped a higher quantity of impurities during the performed chemical recycling process.

In the end, the chemical characterizations were carried out by means of Py-GC/MS, MALDI-TOF-MS and FT-IT ATR. The latter allowed for investigating how the recycled polymer’s structure changed, starting from the initial epoxy systems.

These findings paved the way for further investigation of re-use strategies for the obtained recycled products, with the aim to assess an effective C2C approach in line with a circular economy (CE) philosophy.

## Figures and Tables

**Table 1:** Formulated epoxy resin systems.

Sample ID	PolarBear [%wt]	Epoxidized Waste Flour [%wt]	Recyclamine^TM^ R*101 [phr]
EWF50_A15	50	50	15
EWF100_A22	0	100	22

**Table 2:** The structure of the molecule assigned to peaks of the chromatogram reported in Figure 6.

Peak	Structure	Name
a	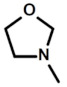	Oxazolidine, 3-methyl-
b	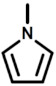	1H-Pyrrole
c	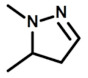	2-Methylperhydro-1,3-oxazine
d	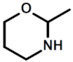	1H-Pyrazole
1	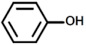	Phenol
2	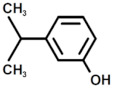	Phenol, 3-(1-Methylethyl)
3	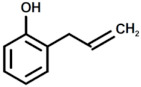	Phenol, 2-(2-Propenyl)
4	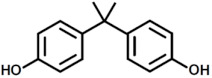	Bisphenol A
5	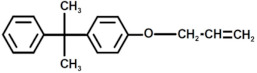	Bisphenol A derivate 1
6	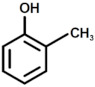	2-methyl phenol
7	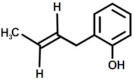	2-(But-2′-Enyl)Phenol
8	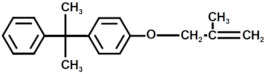	Bisphenol A derivate 2
9	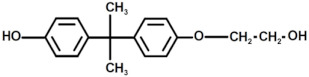	Bisphenol A derivate 3
10	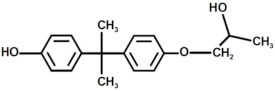	Bisphenol A derivate 4

## Data Availability

Data are available on request.

## Supplementary Materials

The following supporting information can be downloaded at https://www.mdpi.com/article/10.3390/polym17030335/s1. Figure S1: Pyrolysis-GC/MS acquired chromatogram of epoxy system EWF100_A22 decomposition in helium.; Figure S2: Pyrolysis-GC/MS acquired chromatogram of epoxy system rEWF100_A22 decomposition in helium.
