# Supplementary material for: Chemical Recycling of Bio-Based Epoxy Matrices Based on Precursors Derived from Waste Flour: Recycled Polymers Characterization
Source: Polymers (Basel). 2025 Jan 26;17(3):335. doi: 10.3390/polym17030335 (PMC11821098; doi:10.3390/polym17030335)
Supplement: Supplementary file 1 [file polymers-17-00335-s001.zip › polymers-3433797-supplementary.pdf]

polymers

MDPI

Article

Chemical Recycling of Fully-Recyclable Bio-Based Epoxy Matrices based on Epoxidized Precursors derived from Waste Flour: Chemical and Thermo-mechanical Characterization of the Recycled Polymers

Lorena Saitta [1,*], Sandro Dattilo [2,*], Giuliana Rizzo [1], Claudio Tosto [1], Ignazio Blanco [1], Francesca Ferrari [3], Gloria Anna Carallo [3], Fabrizio Cafaro [3], Antonio Greco [3] and Gianluca Cicala [1]

[1] Department of Civil Engineering and Architecture, University of Catania, Via Santa Sofia 64, Catania, 95125, Italy; lorena.saitta@unict.it; giuliana.rizzo@unict.phd.it; claudio.tosto@unict.it; gianluca.cicala@unict.it
[2] CNR-IPCB, Via Paolo Gaifami 18, 95126 Catania, Italy; sandro.dattilo@cnr.it
[3] Department of Engineering for Innovation, University of Salento, Via per Monteroni, Lecce, 73100, Italy; francesca.ferrari@unisalento.it; gloriaanna.carallo@unisalento.it; fabrizio.cafaro@unisalento.it; antonio.greco@unisalento.it
* Correspondence: lorena.saitta@unict.it; sandro.dattilo@cnr.it

Abstract: This study aims to investigate the chemical recycling of two different fully-recyclable bio-based epoxy matrices based on epoxidized precursors derived from waste flour. The key for their recyclability relies on the use of cleavable hardener. In fact, the latter contains a ketal group in its chemical structure, which is cleavable in mild acetic conditions, so allowing for the breakage of the cured network. The recyclability was successfully assessed for both the two investigated formulations, with a recycling process yield ranging from 80 up to 85%. The recycled polymers presented a T_g up to 69.0 ± 0.4°C, determined by mean of DMA and DSC analysis. Next, the TGA revealed that the thermal decomposition of the specimens primarily occurred around 320°C, attributed to the breaking of C–O and C–N bonds in crosslinked networks. In the end, the chemical characterizations were carried out by mean of Py-GC/MS, MALDI-TOF-MS and FT-IT ATR. In fact, these analysis allowed to investigate how the recycled polymer's structure changed, starting from the initial epoxy systems. This insights on their chemical structure could further allow to identify re-use strategies in accordance with a circular economy approach.

Keywords: recyclable thermosets; circular economy; chemical recycling; bio-based epoxy resins; organic waste; bio-content; green epoxidation

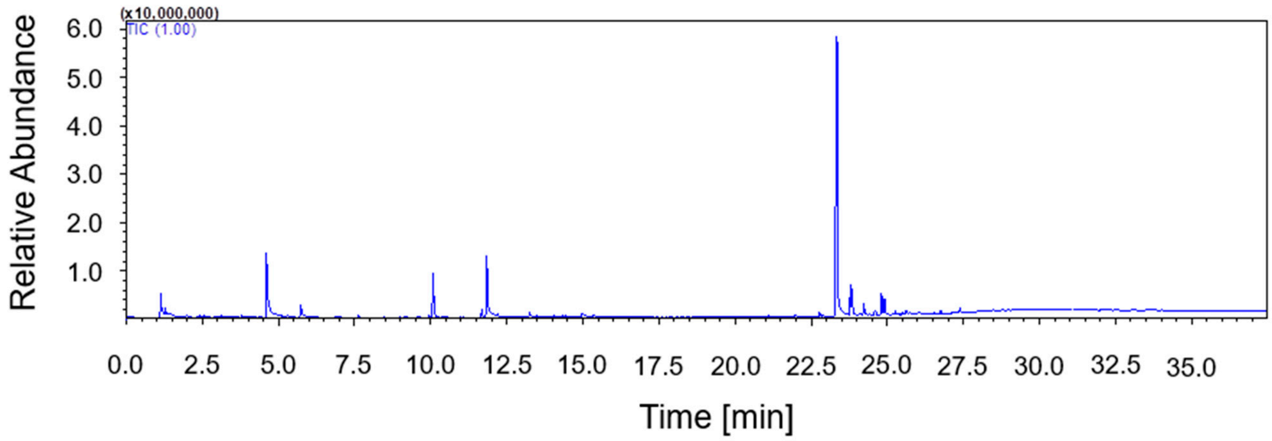

Figure S1. Pyrolysis-GC/MS acquired chromatogram of epoxy system EWF100_A22 decomposition in helium.

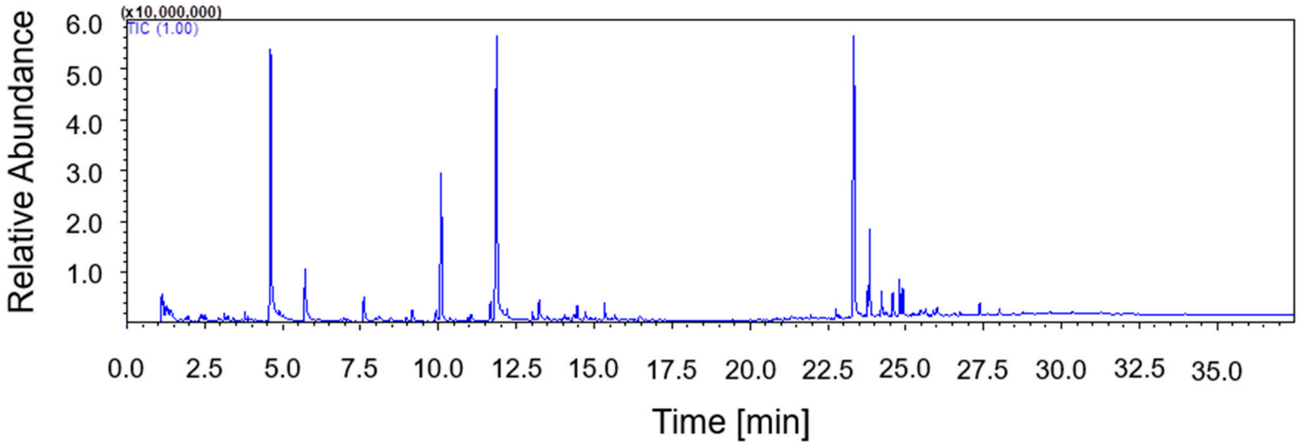

Figure S2. Pyrolysis-GC/MS acquired chromatogram of epoxy system rEWF100_A22 decomposition in helium.
